# POLQ identifies a better response subset to immunotherapy in muscle‐invasive bladder cancer with high PD‐L1

**DOI:** 10.1002/cam4.6962

**Published:** 2024-03-08

**Authors:** Ge Liu, Kaifeng Jin, Zhaopei Liu, Xiaohe Su, Ziyue Xu, Bingyu Li, Jingtong Xu, Yuan Chang, Yiwei Wang, Yu Zhu, Le Xu, Jiejie Xu, Zewei Wang, Hailong Liu, Weijuan Zhang

**Affiliations:** ^1^ NHC Key Laboratory of Glycoconjugate Research, Department of Biochemistry and Molecular Biology, School of Basic Medical Sciences Fudan University Shanghai China; ^2^ Department of Urology, Zhongshan Hospital Fudan University Shanghai China; ^3^ Department of Urology Fudan University Shanghai Cancer Center Shanghai China; ^4^ Department of Immunology, School of Basic Medical Sciences Fudan University Shanghai China; ^5^ Department of Urology, Shanghai Ninth People's Hospital Shanghai Jiao Tong University School of Medicine Shanghai China; ^6^ Department of Urology, Ruijin Hospital Shanghai Jiao Tong University School of Medicine Shanghai China; ^7^ Department of Urology, Xinhua Hospital Shanghai Jiao Tong University School of Medicine Shanghai China

**Keywords:** immunotherapy, muscle‐invasive bladder cancer, PD‐L1, platinum‐based chemotherapy, POLQ

## Abstract

**Background:**

Though programmed cell death‐ligand 1 (PD‐L1) has been used in predicting the efficacy of immune checkpoint blockade (ICB), it is insufficient as a single biomarker. As a key effector of an intrinsically mutagenic microhomology‐mediated end joining (MMEJ) pathway, DNA polymerase theta (POLQ) was overexpressed in various malignancies, whose expression might have an influence on genomic stability, therefore altering the sensitivity to chemotherapy and immunotherapy.

**Methods:**

A total of 1304 patients with muscle‐invasive bladder cancer (MIBC) from six independent cohorts were included in this study. The Zhongshan Hospital (ZSHS) cohort (*n* = 134), The Cancer Genome Atlas (TCGA) cohort (*n* = 391), and the Neo‐cohort (*n* = 148) were included for the investigation of chemotherapeutic response. The IMvigor210 cohort (*n* = 234) and the UNC‐108 cohort (*n* = 89) were used for the assessment of immunotherapeutic response. In addition, the relationship between POLQ and the immune microenvironment was assessed, and GSE32894 (*n* = 308) was used only for the evaluation of the immune microenvironment.

**Results:**

We identified POLQ^high^ PD‐L1^high^ patients could benefit more from immunotherapy and platinum‐based chemotherapy. Further analysis revealed that high POLQ expression was linked to chromosome instability and higher tumor mutational burden (TMB), which might elicit the production of neoantigens. Further, high POLQ expression was associated with an active tumor immune microenvironment with abundant infiltration of immune effector cells and molecules.

**Conclusions:**

The study demonstrated that high POLQ expression was correlated with chromosome instability and antitumor immune microenvironment in MIBC, and the combination of POLQ and PD‐L1 could be used as a superior companion biomarker for predicting the efficacy of immunotherapy.

## Graphical abstract

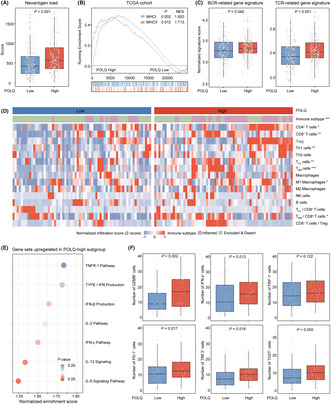

## 1 INTRODUCTION

Approximately 25% of newly diagnosed bladder cancer are muscle‐invasive bladder cancer (MIBC), a highly heterogeneous tumor with high morbidity and mortality.^1^, ^2^ Despite the application of platinum‐based chemotherapy in MIBC, its effectiveness is limited due to the emergence of both de novo and acquired resistance.^3^ The introduction of immune checkpoint blockade (ICB) has changed the treatment landscape of MIBC.^4^ While tumor mutational burden (TMB), CD8 expression, and other immune gene signatures have proven valuable for identifying patients likely to respond to ICB, their applicability is restricted to specific contexts, and further research is required.^5^, ^6^, ^7^ Detection of immune cell programmed cell death‐ligand 1 (PD‐L1) has been confirmed by the Food and Drug Administration (FDA) to be a valid biomarker for response to atezolizumab in patients with metastatic urothelial carcinoma.^8^, ^9^ However, it is insufficient as a single biomarker and there is a need for a comprehensive multiparameter approach.^10^

Failure to accurately repair DNA damage often results in genomic instability, which is a recognized hallmark of cancer.^11^ The defects in DNA damage repair (DDR) can not only impact tumor predisposition but also alter the sensitivity to chemotherapy^12^ and PD‐1/PD‐L1 blockade.^13^ For instance, mutations in genes associated with DNA repairs such as ATM, RB1, and FANCC predict clinical benefit to platinum‐based chemotherapy in MIBC.^14^ POLQ‐associated microhomology‐mediated end joining (MMEJ) pathway could be employed in the presence of DNA damage to compensate for a defective high‐fidelity homologous recombination (HR) repair.^15^ However, as an error‐prone, specialized DNA polymerase, POLQ always produces point mutations and random insertions and deletions at microhomology sites, therefore making MMEJ an intrinsically mutagenic pathway and leading to the rearrangements of the chromosome.^16^ Previous studies have shown that POLQ is upregulated in a wide range of human cancers including colorectal, lung, and breast, along with poor clinical outcomes.^17^, ^18^, ^19^ The clinical values of POLQ in MIBC are worthy of further exploration.

In this study, we demonstrated that in MIBC, POLQ expression could improve the efficacy of PD‐L1 in predicting response to immunotherapy, meanwhile, the combination of POLQ and PD‐L1 could also predict superior response to platinum‐based chemotherapy.

## 2 MATERIALS AND METHODS

### 2.1 Study cohorts

**FIGURE 1:**
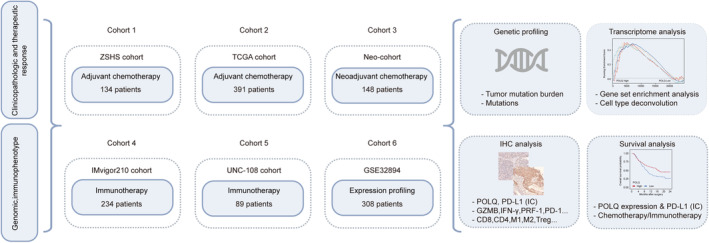
Flow chart of cohort selection and schematic outline of the study.

Six independent cohorts of 1304 MIBC patients were analyzed in this study including the Zhongshan Hospital (ZSHS) cohort, The Cancer Genome Atlas (TCGA) cohort, the IMvigor210 cohort, the Neo‐cohort, the UNC‐108 cohort, and GSE32894 (Figure 1).

The ZSHS cohort involved 215 patients who underwent radical cystectomy from 2002 to 2014 at Zhongshan Hospital, Fudan University. A total of 81 patients were excluded due to non‐urothelial carcinoma (*n* = 13), non‐MIBC (NMIBC, *n* = 60), and tissue detachment on the tissue microarray (TMA, *n* = 8). Therefore, we finally enrolled 134 patients in the ZSHS cohort. Informed consent was obtained from all patients, and the study was approved by the Clinical Research Ethics Committee of Zhongshan Hospital.

The TCGA cohort involved 412 patients from TCGA database. A total of 21 patients were excluded due to lack of follow‐up information or RNA sequencing data (*n* = 7), NMIBC pathologic diagnoses (*n* = 4), or receiving preoperative therapy (*n* = 10). Finally, the TCGA cohort consists of 391 patients.

The IMvigor210 cohort consists of 348 patients from the IMvigor210 trial,^20^ a single‐arm phase II study aimed to investigate the clinical effect of atezolizumab in patients with locally advanced or metastatic urothelial carcinoma. A total of 114 patients were excluded due to lack of TMB data (*n* = 76) or ICB response data (*n* = 38). Both expression data and relevant clinical data in the IMvigor210 cohort were downloaded from http://research‐pub.gene.com/IMvigor210CoreBiologies/.

The UNC‐108 cohort involved 109 patients who received at least one dose of anti‐PD‐1 or anti‐PD‐L1 for advanced urothelial carcinoma.^21^ Only patients with RNA‐sequencing and available ICB response data were included in this study (*n* = 89).

The Neo‐cohort^22^ involved 149 patients who received preoperative cisplatin‐based neoadjuvant chemotherapy before radical cystectomy, and one patient was excluded due to lack of overall survival (OS) data. Finally, we enrolled 148 patients, with 124 receiving neoadjuvant and 24 receiving induction chemotherapy. Additional clinicopathological characteristics of the cohorts included are listed in Tables S1 and S2.

The gene expression matrix GSE32894 from the Gene Expression Omnibus (GEO) database involved 308 patients.

### 2.2 Processing of transcriptomic and genomic data

For transcriptomic data, the mRNA sequencing data were acquired along with the acquisition of clinical information. We used Fragments Per Kilobase of transcript per Million mapped reads (FPKM) to calculate the expression of genes, and normalized mRNA expression by log_2_(FPKM+1) before analysis. Gene signature scores were calculated by the mean arithmetic value of log_2_(FPKM+1) based on the related gene set expression that was reported previously (detailed gene lists are listed in Table S3).^23^, ^24^, ^25^, ^26^, ^27^ The involved genes for gene set enrichment analysis (GSEA) were downloaded from https://gsea‐msigdb.org (detailed sources of GSEA pathways were listed in Table S4). The absolute fraction of 22 types of immune cells was evaluated by CIBERSOFT approach.^28^ Decomposition of 30 cosmic mutational signatures was performed using “deconstructSigs” package. DDR pathway was considered altered if at least one gene in the pathway altered, defined as any non‐silent mutations, including missense, nonsense, insertion, deletion, and splice mutations.^29^ The detailed profiles of core genes involved in HR pathway are listed in Table S3. The total non‐silent somatic mutation per megabase (mut/Mb), which was defined as TMB, was acquired from https://portal.gdc.cancer.gov/.

### 2.3 Immunohistochemistry and assay methods

The immunohistochemistry (IHC) was performed as described previously.^30^ Digital images of TMAs were scanned by NanoZoomer‐XR (Hamamatsu) and Image‐Pro Plus 6.0 under high‐power magnification field (HPF, ×200 magnification). POLQ expression score was obtained by multiplying cellular staining intensity and the proportion of stained cells on a scale of 0–3 by two pathologists who were blinded to the clinicopathological data. The cellular staining intensity was stratified into four degrees: 0 (negative staining), 1 (weak staining), 2 (moderate staining), and 3 (strong staining), and the proportion of stained cells referred to the percentage of positive cells (Figure S1). PD‐L1 expression on immune cells (ICs) was evaluated by IHC using PD‐L1 antibody (SP142) in the ZSHS cohort. Scoring criteria designated tumors as IC0, IC1, or IC2+ (PD‐L1 expression<1%; ≥1% and <5%; ≥5%). Since the IC data were not available in the UNC‐108 cohort, TCGA cohort, and the Neo‐cohort, we defined the top 38.5% as high PD‐L1 mRNA expression with reference to the IMvigor210 cohort (IC2+ accounts for 38.5%). Finally, we divided patients into four subgroups according to the expression of POLQ and PD‐L1. Antibodies used for immunostaining are listed in Table S5.

### 2.4 Statistical analysis

Statistical *p* values were conducted using two‐way tests and detailed statistical tests were shown in corresponding figure legends. Kaplan–Meier curves were constructed to compare OS between different subgroups, whereas log‐rank test and cox regression models were applied for the assessment of the prognostic and risk significance. The Pearson chi‐squared test or Fisher exact test was applied for the analysis of categorical variables, and Mann–Whitney U test was performed for continuous variables. Amplified and deleted regions of chromosome were identified using the Integrative Genomics Viewer 2.14.1.

All statistical analysis were conducted using IBM SPSS Statistics 26.0, GraphPad Prism 8.0.1, GSEA 4.1.0, and R software 4.1.2. *p* ≤ 0.05 was considered statistically significant.

## 3 RESULTS

### 3.1 POLQ^high^ PD‐L1^high^ identifies a subgroup of patients responding to PD‐1/PD‐L1 blockade

**FIGURE 2:**
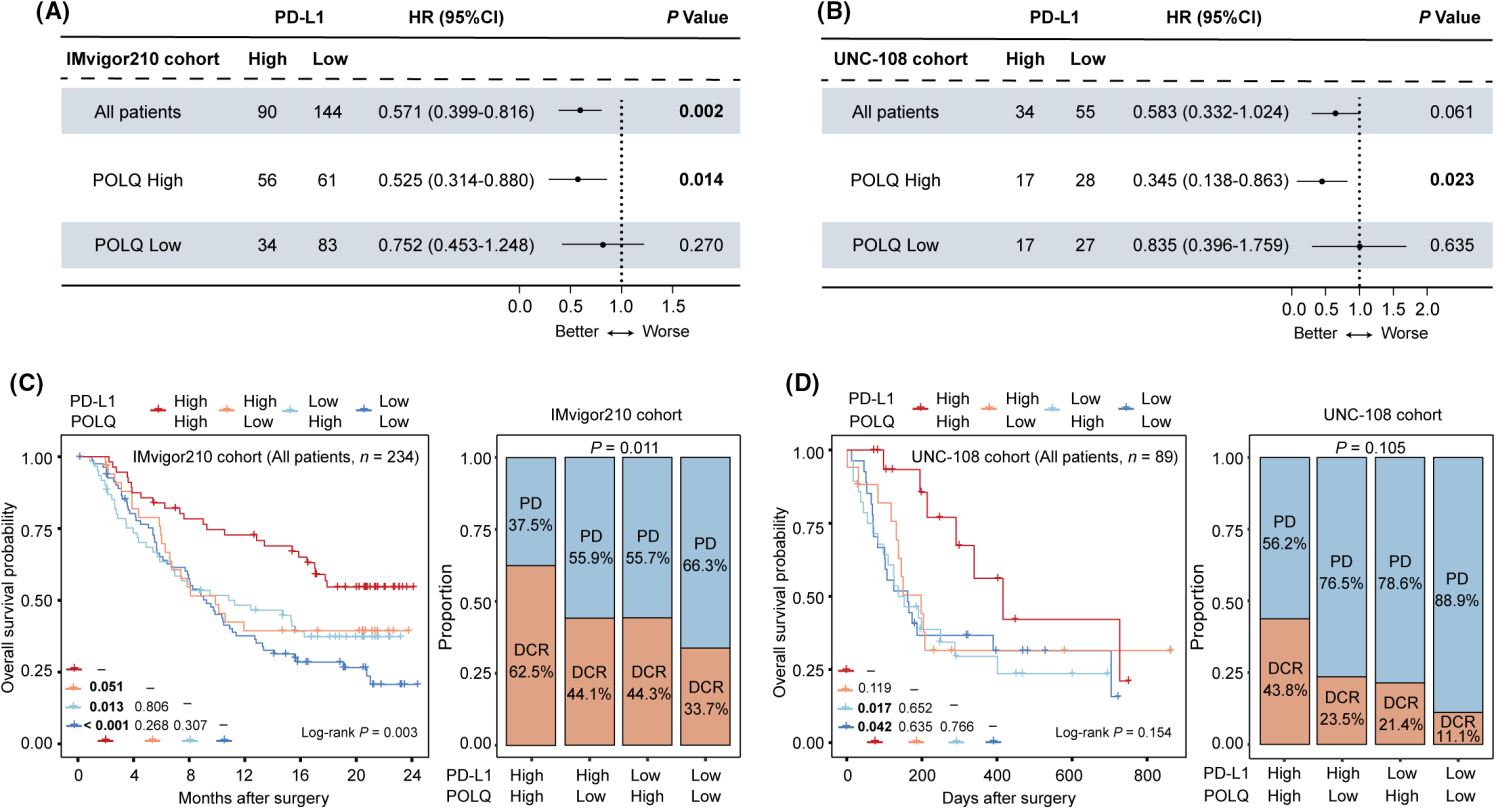
POLQ^high^ PD‐L1^high^ identifies a subgroup of patients responding to PD‐1/PD‐L1 blockade. (A, B) Cox regression analysis of OS according to the expression of PD‐L1 on immune cells among all patients, high POLQ expression subgroup, and low POLQ expression subgroup in the (A) IMvigor210 cohort and (B) UNC‐108 cohort. (C, D) Kaplan–Meier analysis of OS (left) and therapeutic response (right) to PD‐1/PD‐L1 blockade in the (C) IMvigor210 cohort and (D) UNC‐108 cohort, stratified according to POLQ expression and PD‐L1 expression. OS, overall survival; DCR, defined as complete response (CR) + partial response (PR) + stable disease (SD); PD, progressive disease. Pearson's chi‐square test was also applied. *p* ≤ 0.05 was considered statistically significant.

High expression of POLQ and PD‐L1 can indicate better OS to PD‐1/PD‐L1 blockade (Figure S2, *p* = 0.008 for POLQ, *p* = 0.002 for PD‐L1). However, considering the fact that not all PD‐L1‐positive patients respond to PD‐1/PD‐L1 blockade, we first investigated the influence of POLQ stratification on the predictive value of PD‐L1 to PD‐1/PD‐L1 blockade. As displayed in Figure 2A,B, PD‐L1 could predict better clinical outcomes to PD‐1/PD‐L1 blockade in all patients and POLQ^high^ subgroup patients instead of POLQ^low^ subgroup in both the IMvigor210 cohort (*p* = 0.002 for all patients, *p* = 0.014 for POLQ^high^) and the UNC‐108 cohort (*p* = 0.061 for all patients, *p* = 0.023 for POLQ^high^). We considered whether the combination of POLQ and PD‐L1 would distinguish superior responsiveness to PD‐1/PD‐L1 blockade. POLQ^high^ PD‐L1^high^ subgroup showed the best OS and the highest disease control rate (DCR) both in the IMvigor210 cohort and the UNC‐108 cohort (Figure 2C,D and Figure S3A,B). The immune functional signatures including interferon‐γ (IFN‐γ), immune cytolytic, T cell‐inflamed, and T cell‐effector gene signature, which were reported to predict the sensitivity of PD1/PD‐L1 blockade, were elevated in POLQ^high^ PD‐L1^high^ patients (Figure S3C–E). Besides, inflamed immune cells including CD8^+^ T cells and M1 macrophages; effector molecules including granzyme B (GZMB) and IFN‐γ; and immune checkpoints including PD‐1, LAG‐3, and CTLA‐4 were also significantly elevated in POLQ^high^ PD‐L1^high^ patients (Figure S4). These results indicated that the combination of POLQ and PD‐L1 may serve as a better biomarker in predicting the effectiveness of PD‐1/PD‐L1 blockade.

### 3.2 Prognostic value of POLQ alone or in combination with PD‐L1 to platinum‐based chemotherapy

**FIGURE 3:**
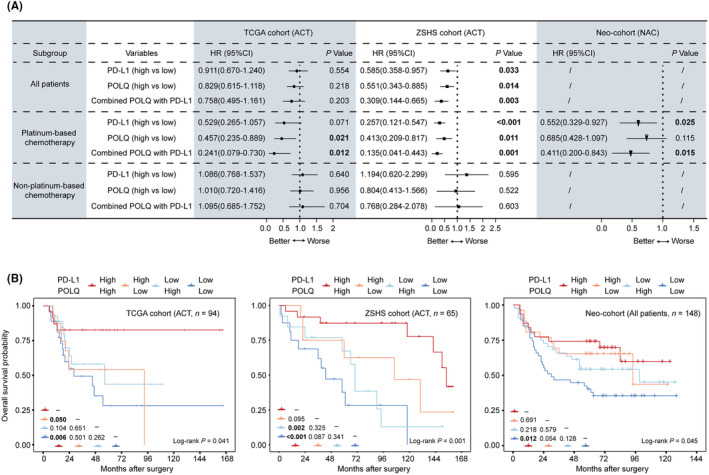
Prognostic value of POLQ alone or in combination with PD‐L1 to platinum‐based chemotherapy. (A) Cox regression analysis of OS according to the expression of POLQ, PD‐L1, and the combination of POLQ and PD‐L1 among all patients, patients with platinum‐based chemotherapy, and patients without platinum‐based chemotherapy in the TCGA cohort, the ZSHS cohort, and the Neo‐cohort. (B) Kaplan–Meier analysis for OS with platinum‐based chemotherapy applied in the TCGA cohort (left, *p* = 0.041), the ZSHS cohort (middle, *p* = 0.001), and the Neo‐cohort (right, *p* = 0.045). HR, hazard ratio; CI, confidence interval; ACT, adjuvant chemotherapy; NAC, neoadjuvant chemotherapy; OS, overall survival. Log‐rank test was conducted for Kaplan–Meier analysis. Cox regression analysis was also applied for survival analysis. *p* ≤ 0.05 was considered statistically significant.

As we confirmed that the combination of POLQ and PD‐L1 could predict better immunotherapy response, we further explored the prognostic value of POLQ, PD‐L1, and their combination to chemotherapy in MIBC. High POLQ expression was associated with better overall survival in patients who received platinum‐based adjuvant chemotherapy from the TCGA cohort (*p* = 0.021, Figure 3A) and the ZSHS cohort (*p* = 0.011, Figure 3A), while no significance was noted in patients who received platinum‐based neoadjuvant chemotherapy from the Neo‐cohort (*p* = 0.115, Figure 3A). Furthermore, the combination of POLQ and PD‐L1 showed superior sensitivity to platinum‐based chemotherapy than POLQ or PD‐L1 alone, in the TCGA cohort (*p* = 0.012, Figure 3A), the ZSHS cohort (*p* = 0.001, Figure 3A), and the Neo‐cohort (*p* = 0.015, Figure 3A). For patients without platinum‐based chemotherapy, there was no evidence that the combination of POLQ and PD‐L1 could serve as a prognostic marker (Figure 3A). Kaplan–Meier curves confirmed that patients in the POLQ^high^ PD‐L1^high^ subgroup possessed better OS than other subgroups among patients with platinum‐based chemotherapy in the TCGA cohort (*p* = 0.041), the ZSHS cohort (*p* = 0.001), and the Neo‐cohort (*p* = 0.045) (Figure 3B). Conclusively, we identified the prognostic significance of POLQ, whether in isolation or in combination with PD‐L1, to platinum‐based chemotherapy.

### 3.3 High POLQ expression is correlated with chromosome instability in MIBC

**FIGURE 4:**
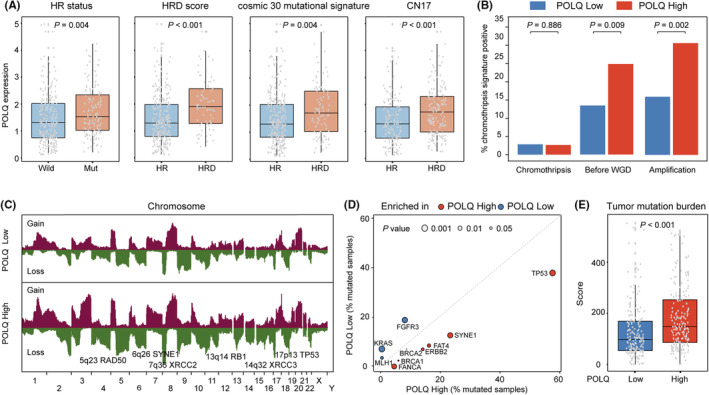
High POLQ expression is correlated with chromosome instability in MIBC. (A) The relationship between POLQ expression and HR deficiency demonstrated by HR mutation status, HRD score, HRD mutational signature, and CN17 (HR feature). (B) The distribution of copy number signatures, including chromothripsis (left), chromothripsis before WGD (middle), and chromothripsis amplification (right) between different POLQ expression subgroups. (C) The association of POLQ expression and copy number alteration (CNA) features. (D) The correlation of POLQ expression and point mutations. (E) The level of TMB in different POLQ expression subgroups. TMB, tumor mutation burden. Data were analyzed by the Mann–Whitney U test and Pearson's chi‐square test. *p* ≤ 0.05 was considered statistically significant.

Considering the DNA repair process conducted by POLQ polymerase has low fidelity, we next evaluated the possible association between POLQ expression and genomic characteristics in MIBC. We observed that POLQ expression is higher in the HR‐deficient subgroup demonstrated by HR mutation status,^29^ HRD score,^31^ HRD mutational signature,^32^ and CN17 (HR feature)^33^ (Figure 4A), which is in accordance with previous reports that the POLQ‐mediated MMEJ pathway is an alternative pathway to compensate for a defective HR repair process. Furthermore, we reported that MIBC patients in POLQ^high^ subgroup possessed a higher level of chromothripsis before WGD and chromothripsis amplification, suggesting the potential association between POLQ expression and chromosome instability (Figure 4B). The analysis of copy number variations (CNVs) between patients with different POLQ expression showed that there were more CNVs in POLQ^high^ subgroup (Figure 4C). Significant gene losses in POLQ^high^ subgroup lay on TP53, SYNE1, and genes related to HR pathway, such as RAD50, XRCC2, and XRCC3 (Figure 4C). Patients in POLQ^high^ subgroup also possessed more mutation in genes such as TP53 (*p* < 0.001), ERBB2 (*p* = 0.037), and SYNE1 (*p* = 0.006), which are potential indicators of response to ICBs or chemotherapy as previously reported (Figure 4D).^34^, ^35^, ^36^ Furthermore, high POLQ expression was related to elevated measures of DNA damage, including the number of segments, the fraction of genome alterations, and aneuploidy (Figure S5). TMB was also higher in patients with high POLQ expression (Figure 4E). Consequently, these findings imply that MIBC patients with high POLQ expression were featured by chromosome instability.

### 3.4 High POLQ expression is correlated with elevated tumor‐specific neoantigens and T‐cell activation

**FIGURE 5:**
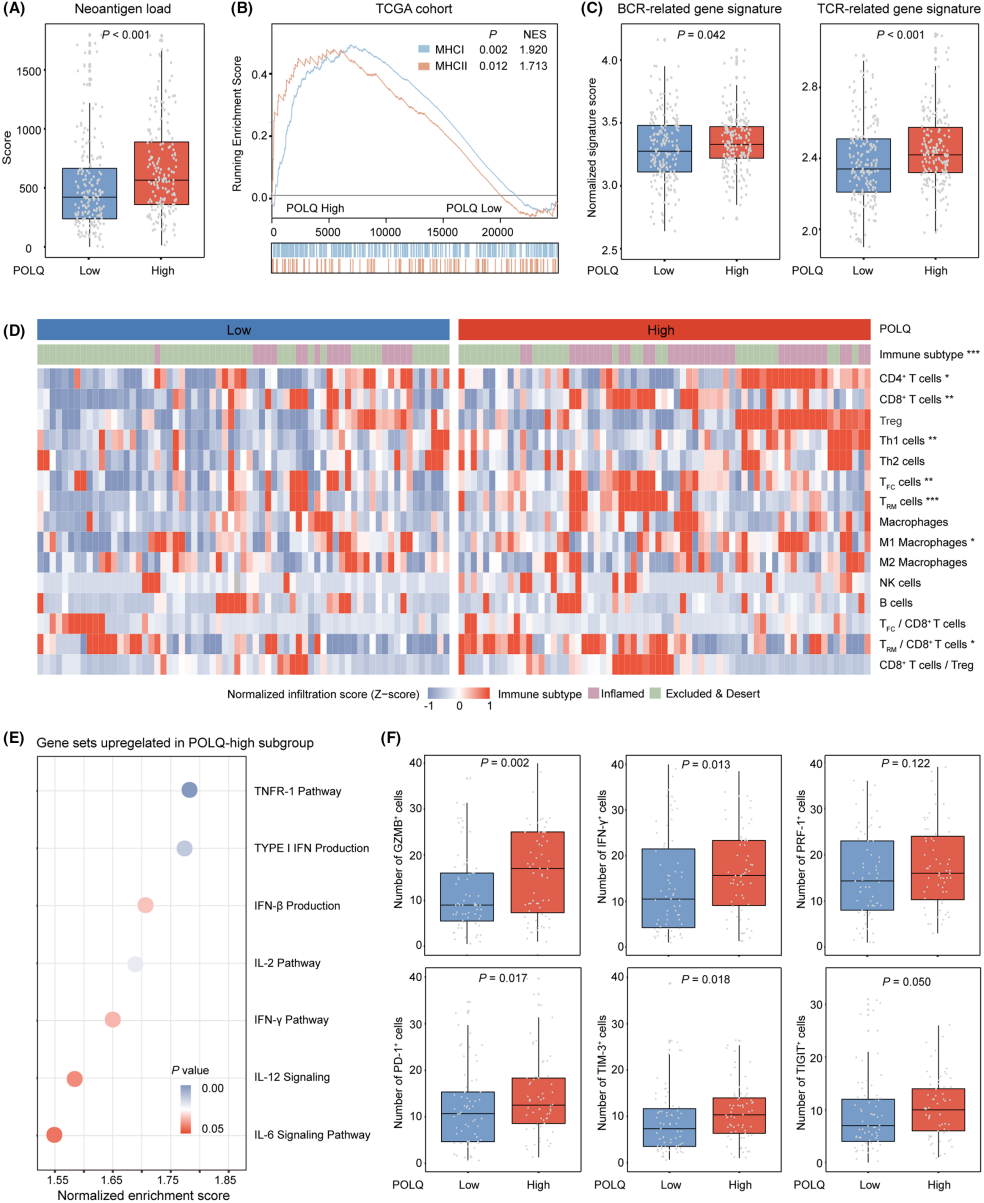
High POLQ expression is correlated with elevated tumor‐specific neoantigens and T‐cell activation. (A) Evaluation of neoantigen load between different POLQ expression subgroups in TCGA cohort. (B) Gene set enrichment analysis (GSEA) to evaluate the antigen‐presenting pathways between different POLQ expression subgroups in the TCGA cohort. (C) Signatures of TCR‐ and BCR‐related pathways between different POLQ expression subgroups in the TCGA cohort. (D) Heatmap of immune cells infiltration between different POLQ expression subgroups in the ZSHS cohort. (E) GSEA to evaluate the immune‐related pathways between different POLQ expression subgroups in the TCGA cohort. (F) Infiltration of effector molecules and immune checkpoint molecules based on POLQ expression in the ZSHS cohort. GZMB, granzyme B; IFN‐γ, interferon‐γ; PRF‐1, perforin‐1. Data were analyzed by the Mann–Whitney U test and Pearson's chi‐square test. *p* ≤ 0.05 was considered statistically significant. *p ≤ 0.05, **p ≤ 0.01, ***p ≤ 0.001.

Given that high POLQ expression correlated with higher genomic instability and elevated TMB, which were thought to result in the generation of tumor‐specific neoantigens that can subsequently be targeted by the immune system,^37^ we next explore the influence of POLQ expression on tumor cell‐intrinsic immunogenicity in MIBC. Initially, we compared the neoantigen load between different POLQ expression subgroups. We observed a higher neoantigen load in the POLQ^high^ subgroup than in the POLQ^low^ subgroup (Figure 5A). MHC class I and MHC class II pathways, which are related to immunogenic neoepitopes presenting, were both hyperactivated in POLQ^high^ subgroup (Figure 5B). Meanwhile, higher levels of T‐cell receptor (TCR) and B‐cell receptor (BCR) gene signatures were also observed in POLQ^high^ subgroup (Figure 5C). We further attempted to depict the immune infiltration between POLQ subgroups. Patients with high POLQ expression in the ZSHS cohort displayed an inflamed immune phenotype, with antitumor immune cells abundance (including CD8^+^ T cells *p* = 0.003, Th1 cells *p* = 0.002, M1 macrophages *p* = 0.020, CD103^+^CD8^+^ tissue‐resident memory T (T_RM_) cells *p* < 0.001, CXCR5^+^CD8^+^ T (T_FC_) cells *p* = 0.003, and T_RM_/CD8^+^ T ratio *p* = 0.022) (Figure 5D). Consistent with the above findings, multiple antitumor cytokine pathways, including IL‐2, IL‐6, interferon‐β (IFN‐β), and IFN‐γ were enriched in patients with high POLQ expression in the TCGA cohort (Figure 5E). Besides, the effector molecules (GZMB^+^ cells *p* = 0.002, IFN‐γ^+^ cells *p* = 0.013) and immune checkpoints (PD‐1^+^ cells *p* = 0.017, TIM‐3^+^ cells *p* = 0.018, and TIGIT^+^ cells *p* = 0.050) were overexpressed in POLQ^high^ subgroup (Figure 5F). Immunotherapeutic signatures in MIBC could only predict clinical OS after PD‐1/PD‐L1 blockade in the whole population and POLQ^high^ subgroup (Figure S6). Collectively, these results demonstrated that high POLQ expression might shape an inflamed microenvironment via the neoantigen‐dependent pathway.

## 4 DISCUSSION

Recent findings propose that patients exhibiting elevated PD‐L1 expression demonstrate enhanced clinical outcomes when subjected to PD‐1/PD‐L1 blockade.^20^, ^27^, ^38^ Consequently, PD‐L1 expression is now regarded as a biomarker for stratifying patients responsive to ICB. Nevertheless, the lack of responses in certain individuals with high PD‐L1 expression and the notable responses in others with low PD‐L1 expression complicate the utility of PD‐L1 as an exclusive predictive biomarker in clinical practice. Therefore, optimizing biomarkers to pinpoint the ideal patient population for ICB is crucial in clinical practice.

In IMvigor210 clinical trials, the use of atezolizumab in the first line did not show a significant improvement in the objective response rate (ORR) with increasing PD‐L1 enrichment.^9^ Additionally, patients overexpressing PD‐L1 (IC2/3) did not get survival benefit from atezolizumab compared to chemotherapy from IMvigor211 clinical trials.^39^ Similarly, in KEYNOTE‐045, treatment benefit was observed regardless of PD‐L1 status.^40^ Regarding the clinical challenge, there is an urgent need to augment the role of PD‐L1 as a predictive biomarker for ICB. Our findings suggest that POLQ expression could assist in identifying MIBC patients with high PD‐L1 expression who are most responsive to ICB.

As a key factor in the MMEJ pathway, POLQ has adverse effects on genomic stability in different tumors. In human laryngeal and cervical cell lines, POLQ has been shown to protect genomic stability,^41^ whereas, in human lung fibroblasts, overexpression of POLQ increases DNA damage markers.^18^

Protects against genomic instability could be attributed to its role in translesion synthesis, which prevents cancer‐induced chromosome rearrangements. Conversely, the potential for destabilization may arise from its role in MMEJ.^15^ We found that in MIBC, patients with high POLQ expression were associated with chromosome instability including chromothripsis, increased CNVs, and elevated measures of DNA damage. A possible explanation for this might be that as a backup choice for DSB repair, MMEJ could be enhanced at the expense of genomic stability when HR is impaired.^42^ Established evidence denotes that elevated POLQ expression was associated with greater mutations in cancer.^16^ Consistent with these findings, we investigated that in MIBC, high POLQ expression was correlated with more mutations in TP53, ERBB2, and SYNE1, which might explain why patients with high POLQ expression are sensitive to ICB or chemotherapy.

Chromosome instability and higher TMB might generate more neoantigens, which can be recognized through the antigen‐processing machinery and MHC molecules.^5^ However, no studies report the effect of POLQ on the immune microenvironment. In this study, we investigated the correlation between POLQ and the immune microenvironment, discovering that individuals exhibiting elevated POLQ expression displayed a T cell‐inflamed microenvironment characterized by the abundance of antitumor immune cells and effector molecules. These findings may account for the responsiveness to immunotherapy.

In addition to improving the efficacy of PD‐L1 in predicting response to immunotherapy, established evidence denotes that POLQ could also serve as a potential target for therapeutic intervention. A recent study found that the antibiotic NVB, a specific inhibitor of POLQ, could kill HR‐deficient breast and ovarian tumors, and enhance the cytotoxic effect of PARP inhibitors.^43^ Another POLQ inhibitor ART558 has been found to cause DNA damage and exhibits synergistic effects when combined with PARP inhibitors in human colorectal cancer cells.^44^ Further, there are 140 POLQ synthetic lethal genes involved in other DDR pathways,^45^ which substantially expand the application of POLQ inhibitors. The first clinical trial (NCT04991480) has recently been carried out to assess the clinical efficacy of POLQ inhibitor ART4215, both as a monotherapy and in combination with other anticancer medications, in patients diagnosed with advanced or metastatic solid tumors. Featured by genomic instability, POLQ inhibitors alone or in combination with immunotherapy are worthy of further clinical investigation in MIBC.

Regardless of the retrospective and exploratory design of our study, these results still require further validation by more extensive, multicentered clinical cohorts. Experimental tests should be carried out to assess the sensitivity of POLQ expression to different DDR‐targeted inhibitors and immune checkpoint inhibitors. Rather, in addition to the neoantigen‐dependent mechanism, POLQ may enhance immune recognition via neoantigen‐independent mechanisms. For example, activation of the STING‐mediated pathway induced by cytosolic DNA fragment accumulation in the setting of DDR deficiency is a potential mechanism that can promote a potent antitumor immune response.^13^, ^46^ Therefore, the mechanism of the relationship between POLQ and antitumor immune response needs further exploration.

In conclusion, our study proposed that both platinum‐based chemotherapy and ICB were suitable options for MIBC patients with high POLQ expression. POLQ^high^ patients represent a specific subtype of MIBC with genomic instability, T cell‐inflamed microenvironment. More importantly, we identified that POLQ may complement immune cell PD‐L1 in selecting patients who are truly responsive to PD‐1/PD‐L1 blockade, which improves the predictive efficacy of presenting ICB‐sensitive biomarkers. These results indicated that adding genomic instability signatures can help optimize personalized therapy.

## AUTHOR CONTRIBUTIONS

**Ge Liu:** Conceptualization (equal); formal analysis (equal); investigation (equal); methodology (equal); validation (equal); visualization (equal); writing – original draft (equal). **Kaifeng Jin:** Conceptualization (equal); methodology (equal); project administration (equal); validation (equal); writing – review and editing (equal). **Zhaopei Liu:** Methodology (equal); project administration (equal); validation (equal); writing – review and editing (equal). **Xiaohe Su:** Methodology (equal); software (equal). **Ziyue Xu:** Methodology (equal); software (equal). **Bingyu Li:** Methodology (equal); software (equal). **Jingtong Xu:** Methodology (equal); software (equal). **Yuan Chang:** Methodology (equal); software (equal). **Yiwei Wang:** Methodology (equal); software (equal). **Yu Zhu:** Methodology (equal); software (equal). **Le Xu:** Methodology (equal); software (equal). **Jiejie Xu:** Conceptualization (equal); data curation (equal); formal analysis (equal); funding acquisition (equal); methodology (equal); resources (equal); supervision (equal); writing – original draft (equal). **Zewei Wang:** Conceptualization (equal); data curation (equal); formal analysis (equal); funding acquisition (equal); methodology (equal); resources (equal); supervision (equal); writing – review and editing (equal). **Hailong Liu:** Conceptualization (equal); data curation (equal); formal analysis (equal); funding acquisition (equal); methodology (equal); resources (equal); supervision (equal); writing – review and editing (equal). **Weijuan Zhang:** Conceptualization (equal); data curation (equal); formal analysis (equal); funding acquisition (equal); project administration (equal); resources (equal); supervision (equal); writing – review and editing (equal).

## FUNDING INFORMATION

This study was funded by grants from the National Natural Science Foundation of China (82002670, 82103408, 82272786, 82272930, 82373276), China Postdoctoral Science Foundation (BX20230091), Shanghai Municipal Natural Science Foundation (22ZR1413400, 23ZR1440300, 23ZR1411700), Shanghai Sailing Program (21YF1407000), Fudan University Shanghai Cancer Center for Outstanding Youth Scholars Foundation (YJYQ201802), and Shanghai Anticancer Association EYAS PROJECT (SACA‐CY22B02, ZYJH202309). All these study sponsors have no roles in the study design, in the collection, analysis, and interpretation of data.

## CONFLICT OF INTEREST STATEMENT

The authors have declared no conflicts of interest.

## ETHICS STATEMENT

This study was approved by the Clinical Research Ethics Committee of Zhongshan Hospital and Fudan University (No. B2015‐030). Written informed consent was obtained from each patient.

## Supporting information


Figure S1.
Figure S2.
Figure S3.
Figure S4.
Figure S5.
Figure S6.
Table S1.
Table S2.
Table S3.
Table S4.
Table S5.

## Data Availability

Data and materials generated that are relevant to the results are included in this article. Other data are available from the corresponding author Prof. Zhang upon reasonable request.
