# Supplementary material for: POLQ identifies a better response subset to immunotherapy in muscle‐invasive bladder cancer with high PD‐L1
Source: Cancer Med. 2024 Mar 8;13(4):e6962. doi: 10.1002/cam4.6962 (PMC10922026; doi:10.1002/cam4.6962)
Supplement: Supplementary file 1 — Figure S1. Figure S2. Figure S3. Figure S4. Figure S5. Figure S6. Table S1. Table S2. Table S3. Table S4. Table S5. [file CAM4-13-e6962-s001.docx]

**POLQ identifies a better response subset to immunotherapy in muscle-invasive bladder cancer with high PD-L1**

**Supplementary Material**

**Supplementary Figure 1. Representative images of immunohistochemistry (IHC) for POLQ expression.**

**Supplementary Figure 2. Predictive value of POLQ and PD-L1 expression to PD-1/PD-L1 blockade.**

**Supplementary Figure 3. POLQ^high^ PD-L1^high^ identifies a subgroup of patients responding to PD-1/PD-L1 blockade.**

**Supplementary Figure 4. Patients with high POLQ expression and high PD-L1 expression are associated with an inflamed immune microenvironment in MIBC.**

**Supplementary Figure 5. Correlation of genome status and POLQ expression.**

**Supplementary Figure 6. Association of POLQ expression and gene signatures validated in clinical studies.**

**Supplementary Table 1. Patient characteristics in the IMvigor210 cohort and the UNC-108 cohort.**

**Supplementary Table 2. Patient characteristics in TCGA, the ZSHS, and the Neo-cohort.**

**Supplementary Table 3. Sources and details of gene signature.**

**Supplementary Table 4. Sources of GSEA pathways.**

**Supplementary Table 5. Immunohistochemistry (IHC) antibodies.**

**
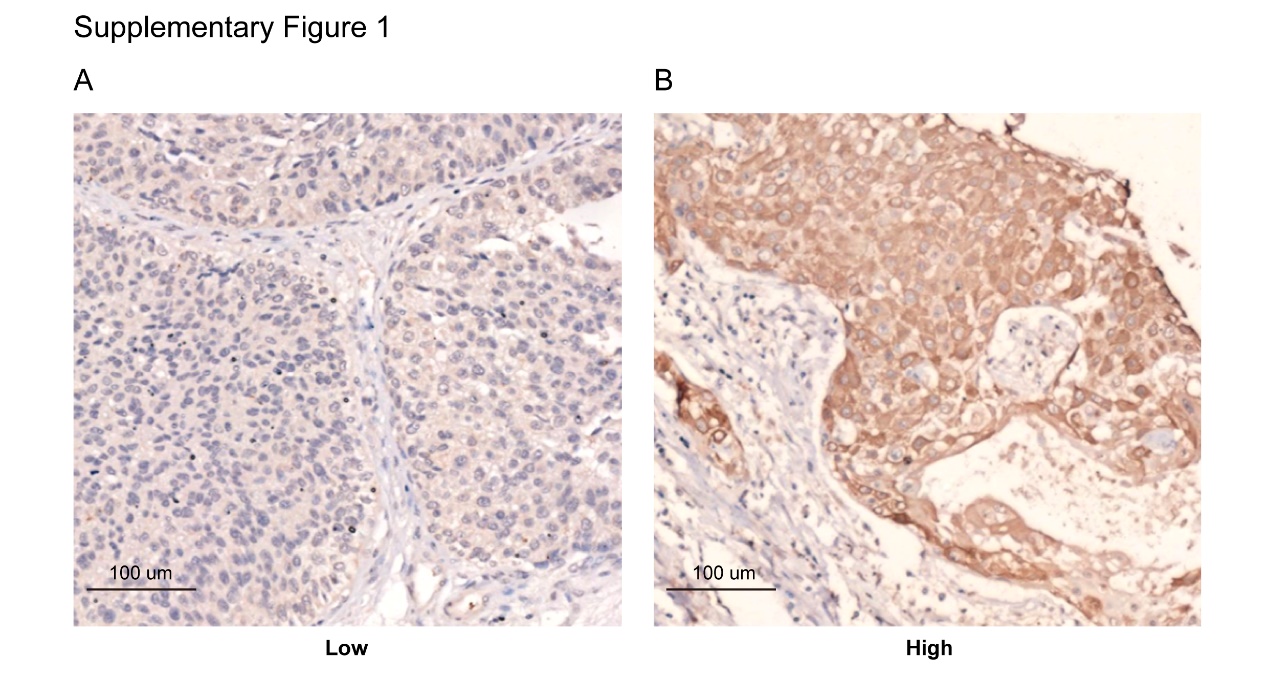
**

**Supplementary Figure 1. Representative images of immunohistochemistry (IHC) for POLQ expression.** (A)MIBC tissues with low staining of POLQ. (B) MIBC tissues with high staining of POLQ. Magnification: 🞨200.

**
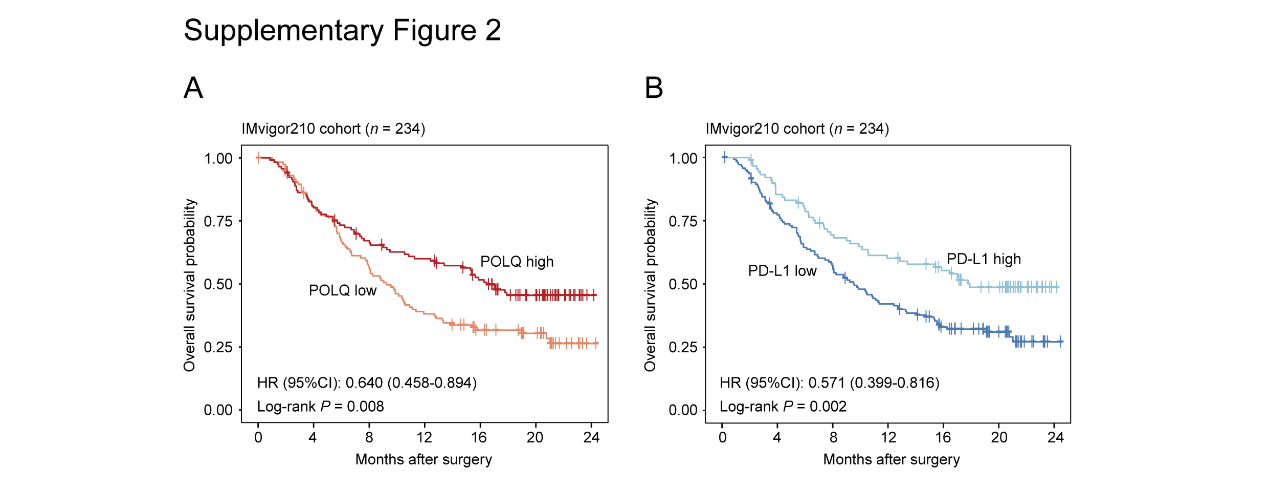
**

**Supplementary Figure 2. Predictive value of POLQ and PD-L1 expression to PD-1/PD-L1 blockade.** (A) Kaplan-Meier analysis for OS to PD-1/PD-L1 blockade in the IMvigor210 cohort according to the POLQ expression [Log-rank *P* = 0.008, HR: 0.640, 95%CI (0.458-0.894)]. (B) Kaplan-Meier analysis for OS to PD-1/PD-L1 blockade in the IMvigor210 cohort according to the PD-L1 expression [Log-rank *P* = 0.002, HR: 0.571, 95%CI: (0.399-0.816)]. HR, hazard ratio; CI, confidence interval; OS, overall survival. Log-rank test was conducted for Kaplan-Meier analysis. *P* ≤ 0.05 was considered statistically significant.

**
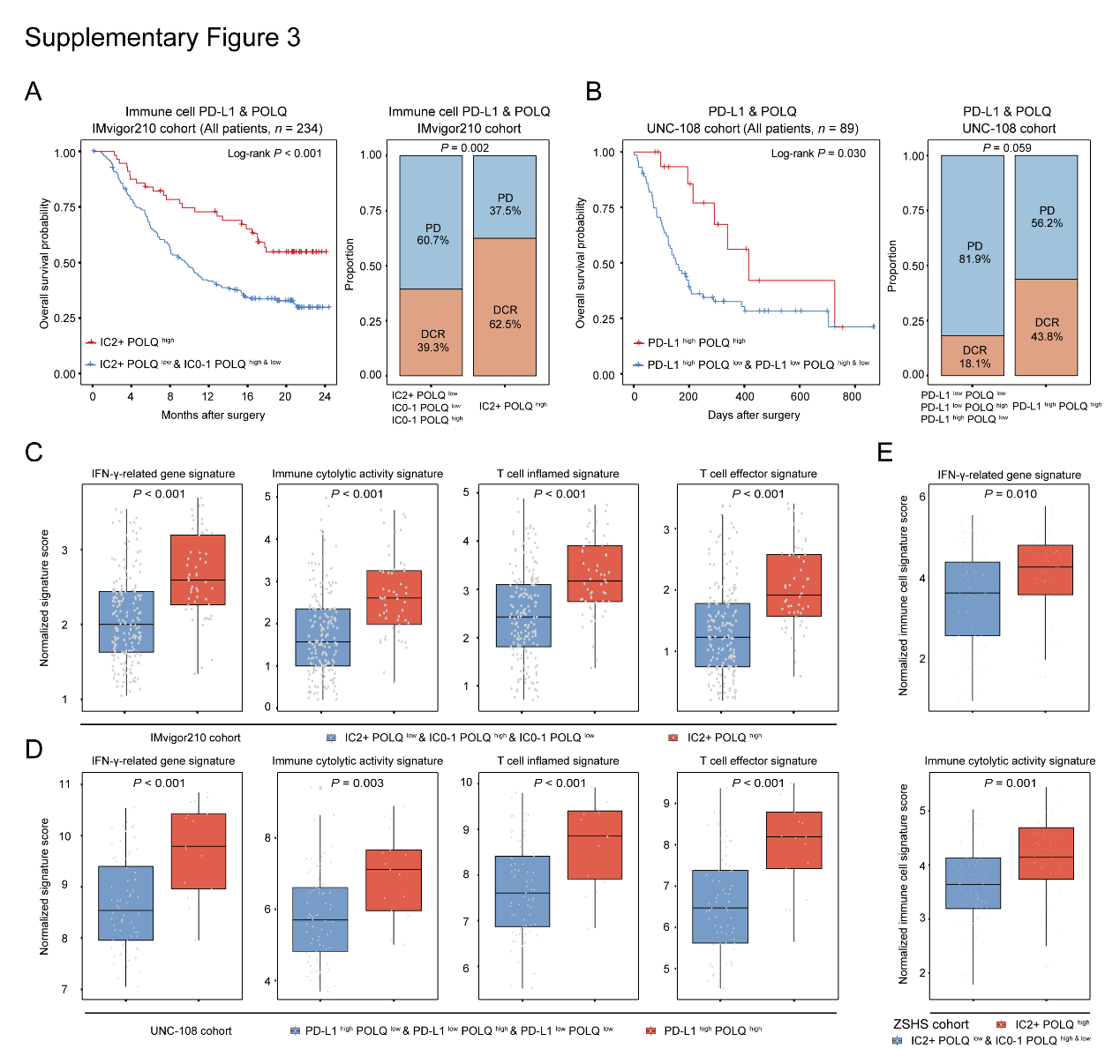
**

**Supplementary Figure 3. POLQ^high^ PD-L1^high^ identifies a subgroup of patients responding to PD-1/PD-L1 blockade.** (A-B) Kaplan-Meier analysis of OS and therapeutic response to PD-1/PD-L1 blockade stratified according to POLQ expression and the PD-L1 immune cell (IC) level in the (A) IMvigor210 cohort and (B) UNC-108 cohort. (C-D) Comparisons of IFN-γ-related gene signature, immune cytolytic activity signature, T cell inflamed signature, and T cell effector signature score between POLQ^high^ PD-L1^high^ subgroup and other combined subgroups in the (C) IMvigor210 cohort and (D) UNC-108 cohort. (E) Comparisons of IFN-γ-related gene signature, and immune cytolytic activity signature between POLQ^high^ PD-L1^high^ subgroup and other subgroups in the ZSHS cohort. Log-rank test was conducted for Kaplan-Meier analysis. Mann-Whitney U test and Pearson’s chi-square test were also applied. *P* ≤ 0.05 was considered statistically significant.

**
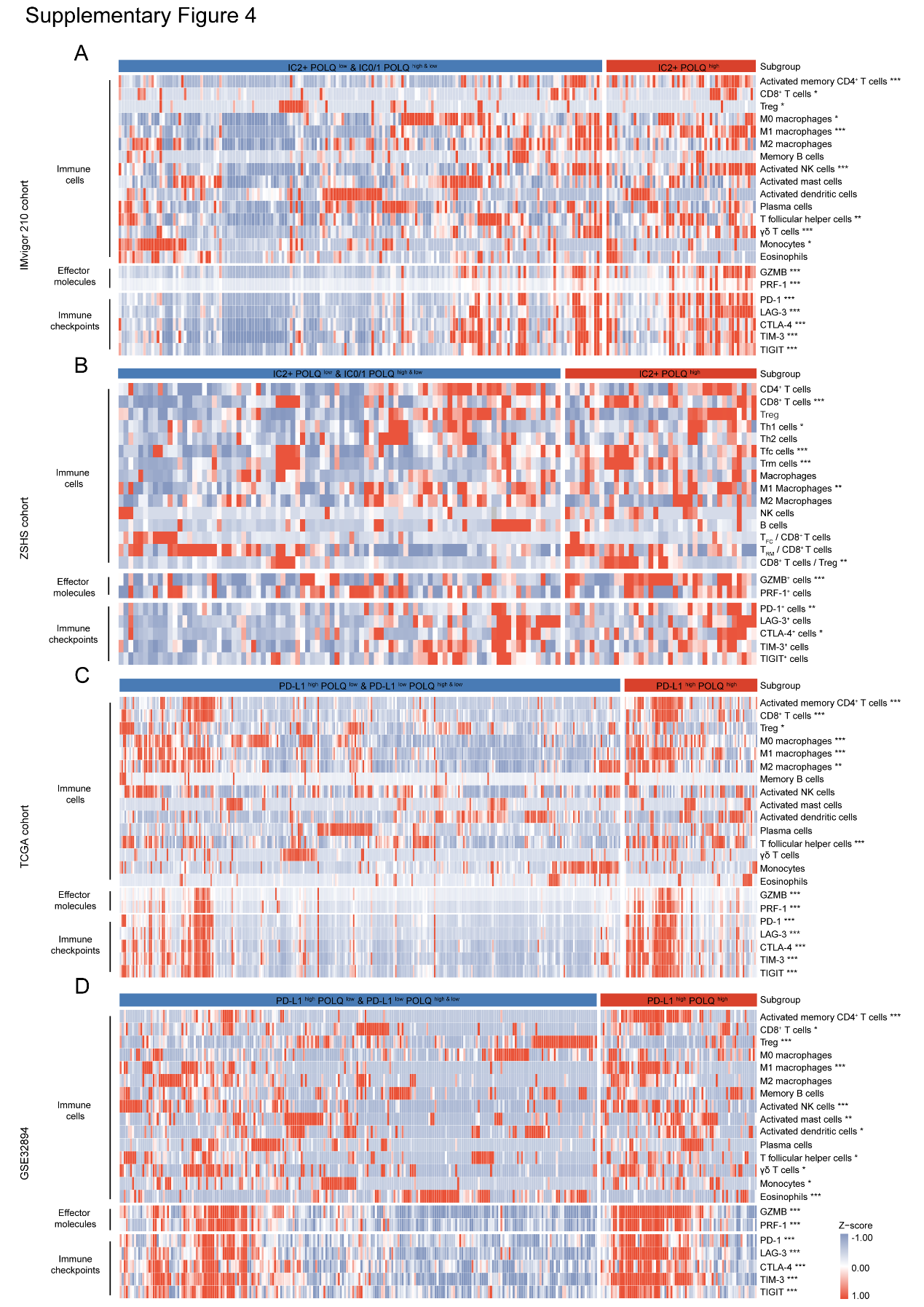
**

**Supplementary Figure 4. Patients with high POLQ expression and high PD-L1 expression are associated with an inflamed immune microenvironment in MIBC.** (A) Heatmap for the infiltration of 22 kinds of immune cells calculated by CIBERSOFT, effector molecules, and immune checkpoints in different subgroups stratified by POLQ and PD-L1 in the IMvigor210 cohort. (B) Heatmap for the infiltration of immune cells, effector molecules, and immune checkpoints in different subgroups stratified by POLQ and PD-L1 in the ZSHS cohort. (C) Heatmap for the infiltration of 22 kinds of immune cells calculated by CIBERSOFT, effector molecules, and immune checkpoints in different subgroups stratified by POLQ and PD-L1 in TCGA cohort. (D) Heatmap for the infiltration of 22 kinds of immune cells calculated by CIBERSOFT, effector molecules, and immune checkpoints in different subgroups stratified by POLQ and PD-L1 in GSE32894. Data were analyzed by the Mann-Whitney U test. *P* ≤ 0.05 was considered statistically significant. **P* ≤ 0.05, ***P* ≤ 0.01, ****P* ≤ 0.001.

**
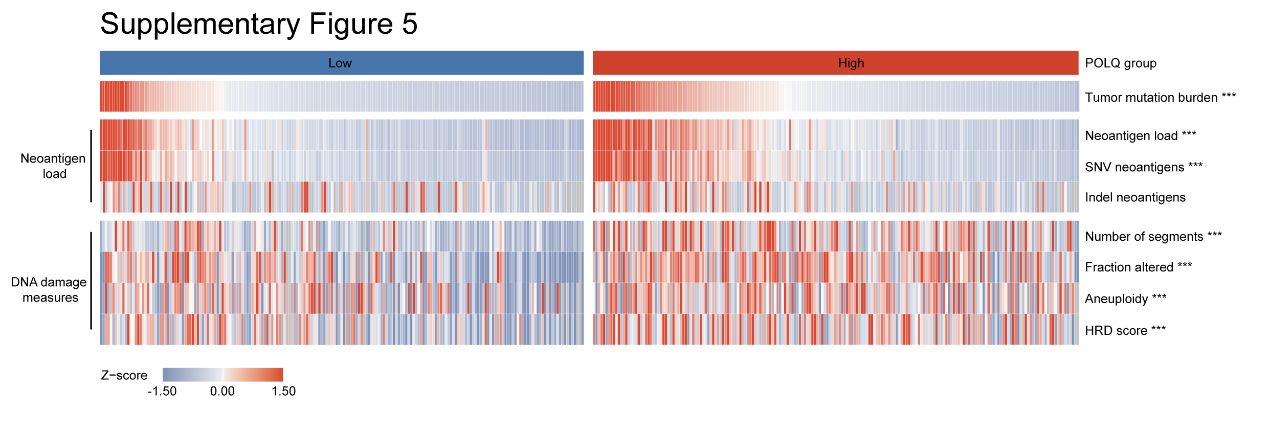
**

**Supplementary Figure 5. Correlation of genome status and POLQ expression.**  Heatmap for the expression of tumor mutation burden, neoantigen load, and measures of DNA damage in POLQ high and low subgroups. Data were analyzed by the Mann-Whitney U test. *P* ≤ 0.05 was considered statistically significant. **P* ≤ 0.05, ***P* ≤ 0.01, ****P* ≤ 0.001.

**
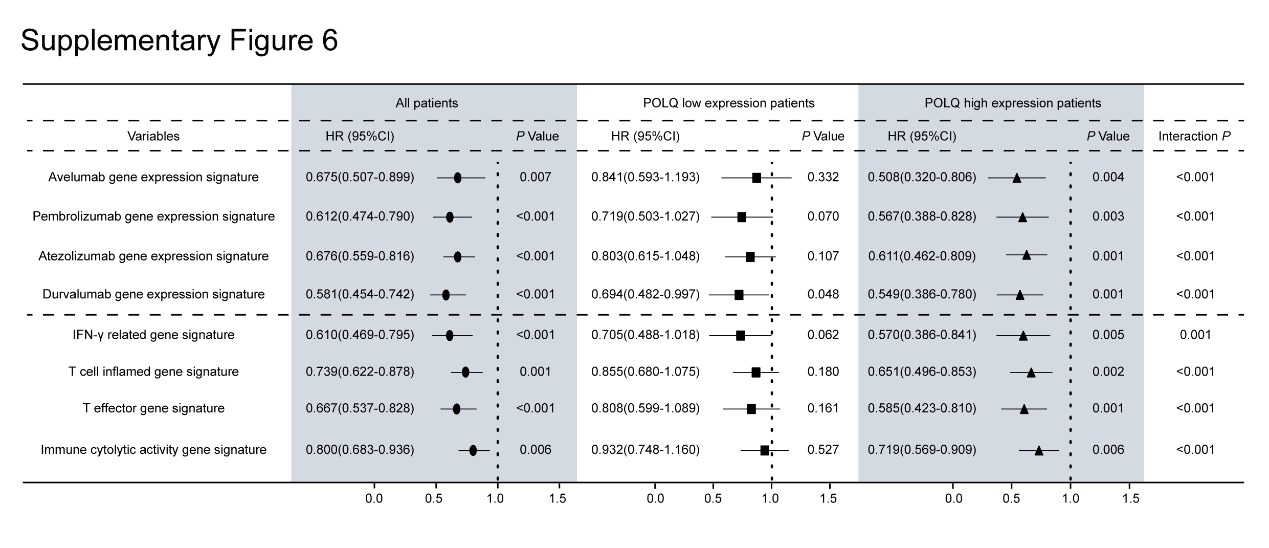
**

**Supplementary Figure 6. Association of POLQ expression and gene signatures validated in clinical studies.** Cox regression analysis of overall survival according to gene signatures validated in clinical studies among all patients, patients with low POLQ expression, and patients with high POLQ expression in the IMvigor210 cohort. Cox regression analysis was applied for survival analysis. *P* ≤ 0.05 was considered statistically significant.

| **Supplementary Table 1. Patient characteristics in the IMvigor210 and the UNC-108 cohort.** | | | | | | | |
| --- | --- | --- | --- | --- | --- | --- | --- |
| **Characteristics** | **IMvigor210 cohort UNC-108 cohort** | | | | | | |
|  | **POLQ expression** | | | **POLQ expression** | | | |
|  | **high** | **low** | ***P*** | | **high** | **low** | ***P*** |
| **Total** | 117 | 117 |  | | 45 | 44 |  |
| **Gender** |  |  | 0.630 | |  |  | 0.603 |
| Male | 91 | 94 |  | | 29 | 26 |  |
| Female | 26 | 23 |  | | 16 | 18 |  |
| **PD-L1^+^ IC Level** |  |  | **0.003** | |  |  | 0.934 |
| IC0/1 | 61 | 83 |  | | 28 | 27 |  |
| IC2+ | 56 | 34 |  | | 17 | 17 |  |
| **PD-L1+ TC Level** |  |  | 0.458 | |  |  | / |
| TC0/1 | 98 | 102 |  | | / | / |  |
| TC2+ | 19 | 15 |  | | / | / |  |
| **TMB** |  |  | **<0.001** | |  |  | 0.055 |
| Low (<10) | 53 | 84 |  | | 31 | 37 |  |
| High (≥10) | 64 | 33 |  | | 14 | 6 |  |
| **Response** |  |  | **0.013** | |  |  | 0.127 |
| PD | 55 | 74 |  | | 31 | 37 |  |
| DCR | 62 | 43 |  | | 13 | 7 |  |
| **Event** |  |  | **0.011** | |  |  | 0.421 |
| Death | 61 | 80 |  | | 27 | 30 |  |
| **Abbreviations：**IC: immune cells; TC: tumor cells; TMB: tumor mutation burden; PD: progressive disease; DCR: Disease control rate, DCR= CR (complete reverse) +PR (partial response) +SD (stable disease).  **P* value was used from Chi-square test; significant *P* value ≤ 0.05 was shown in bold. | | | | | | | |

| **Supplementary Table 2. Patient characteristics in TCGA, the ZSHS, and the Neo-cohort.** | | | | | | | | | | | | | | | | |  |
| --- | --- | --- | --- | --- | --- | --- | --- | --- | --- | --- | --- | --- | --- | --- | --- | --- | --- |
| **Characteristics** | **TCGA cohort ZSHS cohort Neo-cohort** | | | | | | | | | | | | |  |  | |  |
|  | **POLQ expression POLQ expression POLQ expression** | | | | | | | | | | | | | |  | |  |
|  | **high** | **low** | | ***P*** | **high low *P*** | | | | | **high low *P*** | | | | | |  |  |
| **Total** | 196 | 195 | |  | | 67 | 67 |  |  | | 75 | 73 |  | | |  |  |
| **Age** |  |  | 0.444 | | |  |  | 0.284 |  | |  |  | **0.002** | | |  | |
| <60 | 39 | 45 | |  | | 28 | 22 |  |  | | 8 | 23 |  | | |  |  |
| ≥60 | 157 | 150 | |  | | 39 | 45 |  |  | | 67 | 50 |  | | |  |  |
| **Gender** |  |  | 0.975 | | |  |  | 0.162 |  | |  |  | 0.368 | | |  |  |
| Male | 143 | 142 | |  | | 59 | 53 |  |  | | 56 | 59 |  | | |  |  |
| Female | 53 | 53 | |  | | 8 | 14 |  |  | | 19 | 14 |  | | |  |  |
| **AJCC stage** |  |  | 0.652 | | |  |  | 0.669 |  | |  |  | / | | |  |  |
| II | 61 | 64 | |  | | 42 | 41 |  |  | | / | / |  | | |  |  |
| III | 73 | 64 | |  | | 22 | 21 |  |  | | / | / |  | | |  |  |
| IV | 62 | 67 | |  | | 3 | 5 |  |  | |  |  |  | | |  |  |
| **pT stage** |  |  | 0.606 | | |  |  | 0.874 |  | |  |  | / | | |  |  |
| pT2 | 51 | 62 | |  | | 42 | 44 |  |  | | / | / |  | | |  |  |
| pT3 | 96 | 93 | |  | | 15 | 15 |  |  | | / | / |  | | |  |  |
| pT4 | 26 | 30 | |  | | 10 | 8 |  |  | | / | / |  | | |  |  |
| **pN stage** |  |  | 0.549 | | |  |  | 0.718 |  | |  |  | 0.796 | | |  |  |
| pN0 | 117 | 111 | |  | | 64 | 62 |  |  | | 58 | 53 |  | | |  |  |
| pN+ | 59 | 64 | |  | | 3 | 5 |  |  | | 16 | 19 |  | | |  |  |
| pX | / | / | |  | | / | / |  |  | | 1 | 1 |  | | |  |  |
| **Grade** |  |  | **0.001** | | |  |  | 0.819 |  | |  |  | / | | |  |  |
| Low | 3 | 17 | |  | | 12 | 11 |  |  | | / | / |  | | |  |  |
| High | 192 | 177 | |  | | 55 | 56 |  |  | | / | / |  | | |  |  |
| **LVI** |  |  | 0.267 | | |  |  | 0.721 |  | |  |  | / | | |  |  |
| Absent | 71 | 54 | |  | | 26 | 24 |  |  | | / | / |  | | |  |  |
| Present | 71 | 71 | |  | | 41 | 43 |  |  | | / | / |  | | |  |  |
| **ACT** |  |  | 0.389 | | |  |  | 0.167 |  | |  |  | / | | |  |  |
| Applied | 51 | 43 | |  | | 38 | 30 |  |  | | / | / |  | | |  |  |
| Not applied | 144 | 149 | |  | | 29 | 37 |  |  | | / | / |  | | |  |  |
| **Abbreviations：**AJCC: American Joint Committee on cancer; LVI: lymphatic vessel invasion; ACT: adjuvant chemotherapy.  * *P* value was used from Chi-square test; significant *P* value ≤ 0.05 was shown in bold. | | | | | | | | | | | | | | | |  |  |

| **Supplementary Table 3. Sources and details of gene signatures.** | | |
| --- | --- | --- |
| **Signature*** | **Source** | **Genes** |
| IFN-γ-related gene signature | PMID: 28650338 | *CD8A, CCL5, CD27, CD274, PDCD1LG2, CD276, CMKLR1, CXCL9, CXCR6, HLA-DQA1, HLA-DRB1, HLA-E, IDO1, LAG3, NKG7, PSMB10, STAT1, TIGIT* |
| Immune cytolytic activity signature | PMID: 25594174 | *GZMA, PRF1* |
| T cell-inflamed signature | PMID: 25970248 | *IRF1, CD8A, CCL2, CCL3, CCL4, CXCL9, CXCL10, ICOS, GZMK, HLA-DMA, HLA-DMB, HLA-DOA, HLA-DOB* |
| T effector signature | PMID: 25428504 | *GZMA, GZMB, PRF1, EOMES, IFNG, TNF, CXCL9, CXCL10, CD8A, CD4, FOXP3, ICOS, CTLA4* |
| Immune cytolytic activity signature (ZSHS) |  | *GZMB, PRF1* |
| Avelumab gene expression signature | PMID: 34893775 | *EOMES,* *CCL5,* *CD2,* *CD244,* *CD247,* *CD3E,* *CD3G,* *CD6,* *CD8B,* *CD96,* *CST7,* *GFI1,* *GPR18,* *GRAP2,* *IL7R,* *ITK,* *KCNA3,* *KLRD1,* *NLRC3,* *PRF1,* *PSTPIP1,* *SH2D1A,* *SIT1,* *THEMIS,* *TRAT1,* *XCL2* |
| Pembrolizumab gene expression signature | PMID: 34893775 | *CXCL9,* *CD8A,* *CD274,* *LAG3,* *CCL5,* *TIGIT,* *CD27,* *CD276,* *CMKLR1,* *CXCR6,* *HLA-DQA1,* *HLA-DRB1,* *HLA-E,* *IDO1,* *NKG7,* *PDCD1LG2,* *PSMB10,* *STAT1* |
| Atezolizumab gene expression signature | PMID: 34893775 | *CXCL9,* *CD8A,* *IFNG,* *EOMES,* *CXCL10,* *GZMA,* *GZMB,* *TBX21* |
| Durvalumab gene expression signature | PMID: 34893775 | *CXCL9,* *IFNG,* *CD274,* *LAG3* |
| HR pathway | PMID:29617664 | *MRE11A,* *NBN,* *RAD50,* *XRCC2,* *XRCC3,* *BARD1,* *BLM,* *BRCA1,* *BRCA2,* *BRIP1,* *EME1,* *GEN1,* *MUS81,* *RAD51,* *RAD52,* *RBBP8,* *SHFM1,* *SLX1A,* *TOP3A,* *FANCA,* *FANCB,* *FANCC,* *FANCD2,* *FANCL,* *FANCM,* *UBE2T* |

| **Supplementary Table 4. Sources of GSEA pathways.** | |
| --- | --- |
|  | Pathway |
| MHCI | REACTOME_CLASS_I_MHC_MEDIATED_ANTIGEN_PROCESSING_PRESENTATION |
| MHCII | REACTOME_MHC_CLASS_II_ANTIGEN_PRESENTATION |
| BCR | BIOCARTA_BCR_PATHWAY |
| TCR | KEGG_T_CELL_RECEPTOR_SIGNALING_PATHWAY |
| Effector versus memory CD8 T cells-UP | GOLDRATH_EFF_VS_MEMORY_CD8_TCELL_UP |
| B lymphocytes versus CD8 T cells-DN | GSE6259_BCELL_VS_CD8_TCELL_DN |
| Naive versus activated CD8 T cells-DN | GSE15324_NAIVE_VS_ACTIVATED_CD8_TCELL_DN |
| Effective memory CD4 T cells versus Th1 cells-DN | GSE3982_EFF_MEMORY_CD4_TCELL_VS_TH1_DN |
| Naive versus effector CD8 T cells-DN | GSE41867_NAIVE_VS_EFFECTOR_CD8_TCELL_DN |
| Naive versus activated NKT cell-DN | GSE28726_NAIVE_VS_ACTIVATED_NKTCELL_DN |
| Effector versus memory CD8 T cells-UP | GSE9650_EFFECTOR_VS_MEMORY_CD8_TCELL_UP |
| TNFR-1 Pathway | BIOCARTA_TNFR1_PATHWAY |
| TYPE I IFN Production | GOBP_TYPE_I_INTERFERON_PRODUCTION |
| IFN-β Production | GOBP_INTERFERON_BETA_PRODUCTION |
| IL-2 Pathway | BIOCARTA_IL2_PATHWAY |
| IFN-γ Pathway | PID_IFNG_PATHWAY |
| IL-12 Signaling | REACTOME_INTERLEUKIN_12_SIGNALING |
| IL-6 Signaling Pathway | WP_IL6_SIGNALING_PATHWAY |

| **Supplementary Table 5. Immunohistochemistry (IHC) antibodies.** | | | |
| --- | --- | --- | --- |
|  | **Antibodies information** | **Diluted** | **Evaluation** |
| POLQ^+^ cells | Anti-DNA Polymerase theta（Monoclonal; Rb; Abcam; ab111218) | 1:50 | Cell count(200X) |
| CD4^+^ T cells | Anti-CD4 antibody (Monoclonal; Ms; Abcam; ab67001) | 1:50 | Cell count(200X) |
| CD8^+^ T cells | Anti-CD8 alpha antibody (Monoclonal; Ms; Abcam; ab17147) | 1:100 | Cell count(200X) |
| Treg cells | Anti-FOXP3 antibody (Monoclonal; Ms; Abcam; ab22510) | 1:100 | Cell count(200X) |
| Th1 cells | Anti-CD4 antibody (Monoclonal; Ms; Abcam; ab67001) | 1:50 | Cell count (per specimen) |
|  | Anti-T-bet antibody (Monoclonal; Rb; Abcam; ab150440) | 1:500 |  |
| Th2 cells | Anti-CD4 antibody (Monoclonal; Ms; Abcam; ab67001) | 1:50 | Cell count (per specimen) |
|  | Anti-GATA3 antibody (Monoclonal; Rb; Abcam; ab186371) | 1:500 |  |
| Macrophages | Anti-CD68 antibody (Monoclonal; Ms; Abcam; ab955) | 1:400 | Cell count(200X) |
| M1 macrophages | Anti-CD68 antibody (Monoclonal; Ms; Abcam; ab955) | 1:400 | Cell count(200X) |
|  | Anti-HLA-DR antibody (Monoclonal; Rb; Abcam; ab92511) | 1:250 |  |
| M2 macrophages | Anti-MRC1 antibody (Monoclonal; Rb; Sigma; HPA004114) | 1:500 | Cell count(200X) |
| T_FC_ cells | Anti-CXCR5 antibody (Polyclonal; Rb; Abcam; ab46218) | 1:1000 | Cell count(200X) |
|  | Anti-CD8 alpha antibody (Monoclonal; Ms; Abcam; ab17147) | 1:100 |  |
| T_RM_ cells | Anti-CD103 antibody (Monoclonal; Rb; Abcam; ab129202) | 1:300 | Cell count(200X) |
|  | Anti-CD8 alpha antibody (Monoclonal; Ms; Abcam; ab17147) | 1:100 |  |
| B cells | Anti-CD19 antibody (Monoclonal; Ms; Abcam; ab31947) | 1:400 | Cell count(200X) |
| Neutrophils | Anti-CD66b antibody (Polyclonal; Rb; Abcam; ab197678) | 1:1000 | Cell count (per specimen) |
| GZMB^+^ cells | Anti-Granzyme B antibody (Polyclonal; Rb; Abcam; ab4059) | 1:200 | Cell count(200X) |
| IFN-γ^+^ cells | Anti-Interferon gamma antibody (Polyclonal; Rb; Abcam; Ab9657) | 1:300 | Cell count(200X) |
| PRF-1^+^ cells | Anti-Perforin antibody (Monoclonal; Ms; Abcam; ab75573) | Ready-to-use | Cell count(200X) |
| PD-1^+^ cells | Anti-PD1 antibody (Monoclonal; Ms; Abcam; ab52587) | 1:100 | Cell count(200X) |
| PD-L1^+^ cells | Anti-PD-L1 antibody (Monoclonal; Rb; Abcam; ab228462) | 1:500 | Cell count(200X) |
| TIM-3^+^ cells | Anti-TIM3 antibody (Polyclonal; Rb; Abcam; ab185703) | 1:100 | Cell count(200X) |
| TIGIT^+^ cells | Anti-TIGIT antibody (Monoclonal; Rb; Abcam; ab243903) | 1:100 | Cell count(200X) |
